# Candida Endocarditis: A Review of the Pathogenesis, Morphology, Risk Factors, and Management of an Emerging and Serious Condition

**DOI:** 10.7759/cureus.6695

**Published:** 2020-01-18

**Authors:** Sahil Mamtani, Nawar Muneer Aljanabi, Robins P Gupta Rauniyar, Ashu Acharya, Bilal Haider Malik

**Affiliations:** 1 Infectious Diseases Research, Veterans Affairs Medical Center, Lebanon, USA; 2 Internal Medicine, California Institute of Behavioral Neurosciences and Psychology, Fairfield, USA; 3 Surgery, California Institute of Behavioral Neurosciences and Psychology, Fairfield, USA

**Keywords:** c albicans, endocarditis, candidemia, prosthetic heart valves, candidiasis, infective, candida, non candida albicans candida, c parapsilosis, catheter related blood stream infections

## Abstract

Infective endocarditis is a significant healthcare burden due to the associated high mortality and complications. Endocarditis caused by both *Candida albicans (C. albicans)* and non-*Candida albicans Candida* (NCAC) species has been associated with a substantial rise in in-hospital morbidity and mortality. We used the Pubmed database to identify 47 out of 101 articles that had met our inclusion and exclusion criteria. We had put in place a broad inclusion criterion with no age or gender restrictions. These 47 articles included abstracts, 11 review articles, 13 case reports, 10 research articles, 1 clinical trial report, 1 meta-analysis, and other research articles. And they comprehensively cover the pathogenesis, risk factors, and management of infections caused by *C. albicans* and NCAC species in the past 26 years. The articles we scanned provided us with information on different associations in correlation to multiple types of *Candida* species. Here, we discuss the association between *Candida* and endocarditis, a major cause of morbidity and mortality in both *C. albicans* and NCAC. We also present our understanding regarding this interesting association and attempt to address some of the recurring questions.

## Introduction and background

Infective endocarditis is a significant healthcare burden due to the associated high mortality and subsequent complications. According to a prospective cohort study by the International Collaboration on Endocarditis (ICE), the current six-month mortality rate for the condition is approximately 25%. It is an emerging trend throughout the world, in both developing and developed countries, despite the widespread availability of modern diagnostic and treatment methods [1]. The in-hospital mortality rate and readmission rate related to the condition are on a dangerous upward trend [2-4]. Fungal endocarditis accounts for 1-6% of the total endocarditis spectrum. It has a significant association with prosthetic heart valves and devices and a more extended postoperative recovery period. It also increases the potential risk of congestive heart failure, intravenous drug use (IVDU), catheter use, prolonged antibiotic therapy, and many other maladies [5-25]. In particular, the *Candida* species has been associated with a substantial increase in in-hospital morbidity and mortality [5].

Recent literature on non-*Candida albicans Candida* (NCAC) species involving prosthetic devices and hemodialysis catheters have suggested that these could constitute an independent risk factor in candidiasis [26]. There are still many missing pieces, such as the question as to whether the *Candida* species prefer a specific heart valve area compared to non-fungal endocarditis. Also, It is not clear if any of the risk factors, either combined or individually, predisposes the patient to *Candida* endocarditis. In this review, we examine case reports and previous literature reviews of different *Candida* species to ascertain the troubling association between *Candida* and endocarditis with a high level of urgency. Moreover, IVDU and the rise of candidemia are rising and problematic trends to consider [8]. We also discuss the complications of postoperative recovery compared to medical treatment alone in this literature review. *Candida* endocarditis is an emerging disease and a growing health concern globally, especially among the elderly population and immunosuppressed [23]. Additionally, patients needing long-term hospital care with indwelling devices and catheters often suffer from unwanted life-threatening nosocomial infections [10]. Some medications have displayed outcomes outlined in standard guidelines in particular species of *Candidia* and are reviewed in this article [27]. Anatomically, we perceive that location is one of the factors indicated in the invasion [28]. Genetics often plays a significant role in the attack of a host cell [29-31]. We believe the varied pathology will help us understand the divergent ways of NCAC species [32-38]. Finally, we examine the various diagnostic methods, including echocardiography, and management of the disease, including current and changing guidelines in candidiasis treatment [39,40]. We hope that a rigorous look at the facts presented in this review will foster awareness that will contribute to curbing mortality rates associated with the disease.

## Review

The fungal species in *Candida* fall under two categories: *Candida albicans (C. albicans)* and NCAC [30,31]. Most species from the *Candida* genus can be found as healthy skin and mucocutaneous commensals and are often present throughout life. To date, only *C. albicans* and few other NCAC species such as *C. auris, C. glabrata, C. parapsilosis, C. tropicalis, C. guilliermondii, C. krusei, and C. dubliniensis* have known to act as human pathogens involved in diseases [31]. However, the characteristics, morphogenesis, and pathogenesis differ significantly even within the *Candida* species [12,32-34]. Some authors have suggested an association between certain *Candida* species and particular types of patients. For example, *C. dubliniensis* is associated with human immunodeficiency virus (HIV)-positive individuals with oral thrush, whereas *C. parapsilosis* has been associated with patients who undergo transcatheter aortic valve replacement (TAVR), transplant recipients, and patients on parenteral nutrition (PN). Moreover, *C. parapsilosis* has been identified as the second most common species to be isolated from blood culture, the most common being *C. albicans* [35]. 

Morphological differences between *Candida albicans* and *non-Candida albicans Candida* species

**Figure 1:**
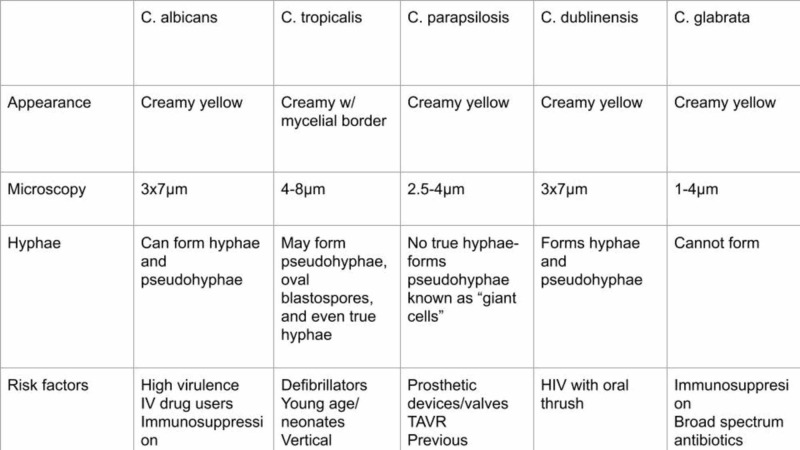
Candida albicans vs. non-Candida albicans Candida species TAVR: transcatheter aortic valve replacement; HIV: human immunodeficiency virus

There are close to 150 heterogenous *Candida* species. However, only a few can survive the normal human body temperature of 37 ºC to establish themselves as human pathogens or commensals. To the naked eye, *Candida* colonies appear creamy yellow and seem to have yeast-like morphology. Microscopically, most species of *Candida* grow in the log phase to approximately 3x7 um in size and look like budding yeasts. Some *Candida* species, such as *C. albicans and C. dubliniensis*, often produce true hyphae or pseudohyphae. Pseudohyphae are elongated projections from parent cells restricted by cell-to-cell junctions. True hyphae, on the other hand, do not display cell-to-cell junctions; instead, they are elongated cells [35]. *C. dubliniensis* is less virulent in comparison to *C. albicans*, leaving less room for an invasion. It has been reported that evolutionary changes in specific gene sequences and the hyphally regulated gene (HYR1) result in decreased pathogenicity of *C. dubliniensis*. The changes in genetic sequences allow us to understand the pertinent factors playing a role in establishing the disease [30]. *C. parapsilosis is* another NCAC species that can form pseudohyphae, which are larger and called "giant cells." [12,27]. *C. tropicalis* displays yeast and pseudohyphal growth in Tween 80 and at 37 ºC for 48 hours [36]. *C. glabrata* is not polymorphic and was not considered a human pathogen earlier. However, due to increasing conditions of immunosuppression and broad-spectrum antibiotic use, *C. glabrata* has emerged as an opportunistic pathogen. Being much smaller in size (around 1-4 um) compared to other pathogenic *Candida* species and being of haploid strain, *C. glabrata *has now become more noticed [26-29,37]. Figure 1 below lays out the differences between the various *Candida* species.

Mode of transmission

Inoculation is different for many of these species. For example, In *C. parapsilosis*, horizontal transmission through infected medical devices, transmission through the hands of healthcare workers, and the use of catheters accounted for a large amount of invasive infection in neonates. In contrast, in *C. albicans and C. tropicalis*, infection in neonates was more frequently through vertical transmission [27]. Fungal invasive infections caused by *C. parapsilosis* often occur through central venous lines and hyperalimentation solutions (parenteral nutrition) due to its increased preference and growth capacity [27,38]. Also, a recent Spanish study of invasive *C. parapsilosis* showed that prolonged vascular catheterization and prior surgery were some of the most critical risk factors [27]. *C. tropicalis* is more commonly associated with malignancy and neutropenia, and consequently with a higher mortality rate [38]. The pathogenesis of certain NCAC species, such as *C. tropicalis*, can also be fatal due to their more elevated virulence-associated markers. For example, biofilm formation and secretion of proteinases in *C. tropicalis* can create an impactful and invasive infection compared to *C. albicans* [27,41]. In a study by Negri et al., *C. tropicalis* adhered more strongly to epithelial cells than silicone, indicating easier dissemination and invasion [41]. 

Colonization

Skin and gastrointestinal (GI) tract colonization is one of the first steps in an invasion [27]. Adherence to biofilm formation can be a step preceding skin and GI colonization and can also involve surfaces of medical devices, other bacteria, and abiotic surfaces. Biofilms are often culprits involved in recurrences and efflux of anti-fungal medications, resulting in relapses and failed therapies. *C. tropicalis* adheres to a defibrillator, causing endocarditis and, after this, a biofilm that formed is a collection of microorganisms within an extracellular gel layer composed of proteins, lipids, and matrix [4,42]. It strengthens the adhesion between host cells or medical devices. Eventually, cells divide, reproduce, and further disseminate, causing widespread infection in different organs by the secretion of hydrolytic enzymes, hemolysins, secreted aspartyl proteases, and lipases. All of these are substantial virulence factors contributing to the widespread diseases associated with *Candida* species [27]. 

Do drug use, hepatitis c, cardiac devices, prosthetic valve implants, and immunocompromised status augment the association between *Candida* and endocarditis?

Despite being a normal human commensal, *Candida* can also switch and transform into a pathogenic yeast. Risk factors are abundant, creating a milieu for a systemic infection affecting the cardiac valves. Of the factors implicated in causation, the following are the ones discussed extensively: IVDU; cardiac devices and prosthetic valve implants; catheter-related infections; end-stage renal disease; hospital-acquired infections; and immunocompromised status. According to a recent case series study on IVDU and *Candida* endocarditis conducted in Massachusetts, there has been a resurgence of injection drug use and infective endocarditis. It is a severe risk factor to consider, as the study claimed that the patients who do IVDU often tend to be younger and have an association with hepatitis C [8]. This study also identified that IVDU patients had lesser incidence of HIV, but a prevalence of hepatitis C, possibly due to the associated increasing rates of hepatitis C in Massachusetts and decreasing rates of HIV. However, this study was limited to a specific region. Further studies might help to identify a stronger connection between hepatitis C and candidemia [8].

Drug Users and Candida Endocarditis

Having additional comorbidity with hepatitis C the complicates the situation of these patients in the setting of an opioid epidemic. Also, the study conducted in Massachusetts reported that *C. albicans* was the third most common cause of infections in the IVDU population in central Massachusetts, preceded by *C. parapsilosis *and* C. glabrata* [8]. This is important since we know that different species of *Candida* are emerging as nosocomial pathogens, which further indicates that increased healthcare management, including the access to catheters, prosthetic valves, and prolonged antibiotic therapies increase the risk [7,12,26-27,29]. However, the findings may also indicate that IVDU in association with *C. parapsilosis *and* C. glabrata* outside of the hospital environment may act as community-acquired pathogens in the wake of a drug epidemic. Another common factor among many patients noted in the case reports and systematic review was diabetes [4,8,23-24,27]. Another vital risk factor was that many patients had either a structural or conduction defect in the heart that was strictly related to *Candida* endocarditis [17]​​​​​.

Other risk factors

The use of prosthetic valves, cardiac devices, and other modern procedures have contributed to the rising incidence of *Candida* endocarditis. An ICE and ICE-Plus trial found that prosthetic valve endocarditis was 50% more common in patients with candidemia when compared to non-fungal endocarditis. The same trial also concluded that patients with a prior history of endocarditis and cardiac surgery were more susceptible to this infection [10]. A 30-year study from 1965 to 1995 provided documented evidence on fungal endocarditis and brought into focus an essential discussion on many risk factors associated with fungal endocarditis [12]. Today, as the population is aging, the reported cases of prosthetic valve involvement, contamination of pacemakers and defibrillators, along with prolonged antibiotic therapy are at their peaks [7,9,12,18,23]. It explains, in part, the rise of C*andida* endocarditis in hospital- and non-hospital-acquired infections. In a majority of the cases, according to one review, endocarditis caused due to fungi often needed readmission. Added to this, injection drug users with a prosthetic valve or prior history of endocarditis are often significantly at risk as repeated injections of pathogens provide a way to cultivate growth on the valves directly [2]. In an observational cohort study based on the ICE trial from 2000 to 2010, congestive heart failure at baseline, persistent candidemia, and heart failure as complications also posed high-risk factors for mortality [17]. Even devices designed as roads to destination or transplantation therapy can be contaminated [20]. However, immunocompetent patients can also develop *Candida endocarditis*. A case report identified a 32-week primigravida who succumbed to mitral valve *Candida endocarditis* despite adequate medical and surgical management. The only known risk factor in her case was an appendicectomy conducted two months prior [21]. Collectively, these risk factors increase in-hospital mortality rates. In consideration of this, a validated risk score was developed, where host factors (hemodialysis), infective endocarditis characteristics (IE) such as prosthetic or nosocomial IE, left-sided endocarditis, and complications of IE such as heart failure, stroke, and persistent bacteremia displayed higher mortality as opposed to surgery for IE [1]. Further, the earlier surgical intervention helped in reducing complications and 6-month death rates [1]. Also, although the ICE study covered many geographic areas and locations where access to multidisciplinary care is available, the quality of care in remote areas with poor access to detection is a limitation [1]. In one study, it was also found that 30-day readmission trends were often high among the uninsured and they often left hospitals against medical advice, leading to delayed treatment and diagnosis [2].

Anatomy 

Anatomically, the most common valves afflicted are the mitral and aortic valves [10,11]. These are valves that often undergo replacement or repair due to a primary aging condition. A study has identified that some type of prior insult to the heart is often present, which in this case are the multiple cardiac surgeries on the aortic, mitral, and tricuspid valves. The patient in this study also had significant immunosuppression (neutropenia) [38]. Another case report stated that *Candida endocarditis *may occur even after a TAVR [22]. Marked obstruction to the flow through the aorta and the extensive vegetations in blood cultures growing *Candida parapsilosis* as the causative agent questions the growing trend of non-*albicans* endocarditis; and the use of new surgical procedures can be detrimental and lead to infective endocarditis [22]. However, the location and association to a particular valve are dependent on a wide array of risk factors. For instance, a case report about *C. albicans* in a 75-years-old female using subcutaneous catheter devices found that there was no valve involvement; it seemed that the right atrial septum was involved [15]. This rare case depicted the propensity of *C. albicans* to implant itself on any location of the endothelium [15]. Another case report supports this theory; this report depicted a foreign body, a fishbone, leading to left atrial *C. albicans* infective endocarditis as a result of its penetration through the esophagus [28]. Another study reported that approximately 16% of central venous catheters grew *Candida* species, causing endocarditis in right-sided valves predominantly [19]. 

Management

An essential tool for diagnosis remains an echocardiogram. Most likely, the lesions appear large and left-sided, often valvular. Transesophageal echocardiography, in contrast to transthoracic, is more sensitive and specific [39]. Guidelines for managing *Candida* endocarditis often incorporate both an urgent surgical and medical approach, in light of the fact that confining the treatment to medical therapy alone may cause embolic attacks, high morbidity, and mortality [39]. Medically, antifungal treatment is often part of the therapy. For example, polyene antifungals such as Amphotericin B create pores in the cell wall, resulting in loss of cytoplasmic content and cell death [27]. Lanosterol demethylase inhibitors such as fluconazole and voriconazole interfere with the synthesis of lanosterol to ergosterol, a key sterol in the cell wall [13,27]. These therapies, in combination with early debridement of all infected tissues, is usually performed to manage *Candida* endocarditis [40]. Further, a meta-analysis of cases from 1966 to 2002 confirmed lower mortality and a prevalence odds ratio (POR) of 0.56 with a 95% confidence interval (CI) of (16, 1.99) in patients receiving combined medical and surgical therapy. In contrast, medical treatment alone contributed to a higher death rate with a POR of 1.49, and 95% CI of (0.39,5.81) [43]. Possible causes of failure of medical treatment alone include relapses due to *Candida* species having the ability to form biofilms, which allow efflux of anti-fungal agents and decrease cell-wall penetration with resultant reduced action [40,43].

However, there are many other challenges to consider in the treatment of *Candida* endocarditis. Some of them include geographic variations, virulence, and reduced susceptibility to antifungal medications [44]. As per the SENTRY Antimicrobial Surveillance Program (2008-2009), there is rising evidence of echinocandin (anidulafungin and micafungin) resistance emerging within *C. glabrata* due to FKS gene mutations [44]. Further, blood culture turnout time may affect earlier detection rates. Therefore, a high clinical suspicion must be maintained in vulnerable populations [45]. In fact, blood cultures are highly variable, providing an antemortem sensitivity rate of 21-71%. This variability hampers the ability of blood cultures to efficiently detect viable cells of *Candida* so that they are quickly eliminated from the circulation, resulting in decreased sensitivity of the test [45]. Moreover, new rapid assays such as mannan and anti-mannan immunoglobulin G (IgG) have a combined sensitivity and specificity of 83 and 86%, respectively [45]. Beta-D glucan assays and polymerase chain reactions (PCR) have been slower to be used for diagnosis but have indicated sensitivity close to 75%. However, these rapid assays may not indicate actual evidence of *Candida;* but they indirectly help in diagnosing *Candida* endocarditis and should be considered earlier to establish a diagnosis [45]. Also, even patients with significant immunosuppression and neutropenia have better rates of detection than blood cultures with these new rapid assays [45]. Patients with neutropenia should also consider echinocandins, such as caspofungin and anidulafungin, as the initial therapy [40]. However, case reports from different institutions suggest management on a case-by-case basis, keeping in mind the severity, prognosis, and autonomy of the patient [2,8,14-16]. For example, according to an Italian study, prolonged antibiotic exposure and abdominal surgery more likely predisposed the patient to develop native valve endocarditis than prosthetic valve endocarditis [46]. Management of *Candida* endocarditis may often involve challenges and may lead to questionable treatment decisions, which may contribute to relapses. Fluconazole, in particular, if given alone, contributes to high relapse rates [40]. However, fluconazole is considered a highly successful antifungal agent for relapses, life-long suppression, and recurrence of prosthetic valve endocarditis [40]. Susceptibility to medical treatment is dependent on the virulence of *Candida* species, which is measured by its ability to secrete aspartic peptidases (Saps) and produce biofilms. A prospective open-label clinical trial has shown the efficacy of caspofungin in the treatment of endocarditis due to its ability to penetrate biofilms [47]. The emergence of new guidelines and data will hopefully help us to easily convey pertinent points and critical risk factors as a guide to evidence-based medicine and contribute to the more efficient management of *Candida* endocarditis.

## Conclusions

A review of the current literature has led us to conclude that there is a significant association in terms of the different risk factors associated with the pathogenesis of different *Candida* species. IVDU happens to be one of the significant risk factors associated with *Candida* endocarditis. Also, patients succumb to complications of congestive heart failure and embolism, and many do not fare well during the post-recovery period. Some solutions include setting up new programs that specialize in the long-term care of patients with *Candida* endocarditis. These programs can help address in-hospital morbidities, rehospitalizations, costs, and mortality. Questions that we propose today have many challenges and need urgent attention. For instance, how do we improve the one and 5-year in-hospital morbidity and mortality rates? What steps should we take to improve the quality control of our institutions to reduce nosocomial infections? How should we detect *Candida* endocarditis in rural areas with poor access to health care? To address these challenges effectively, we should look for solutions collectively as we gain further insight into the association between *Candida* and endocarditis.
